# SEM-ANN analysis of the social prescription of nature, physical activity, and healthy living initiatives for cardiovascular health: mediating role of social support

**DOI:** 10.3389/fpubh.2025.1653515

**Published:** 2025-11-20

**Authors:** Rashid Menhas

**Affiliations:** 1 School of Nursing, Shandong Xiehe University, Jinan, China; 2 Department of Nursing, The Fourth Affiliated Hospital of School of Medicine and International School of Medicine, International Institutes of Medicine, Zhejiang University, Yiwu, China

**Keywords:** CVD, nature, physical activity, healthy living initiative, social prescription, social support

## Abstract

**Background:**

Cardiovascular diseases (CVDs) are a major public health concern, contributing significantly to morbidity and mortality. Several lifestyle factors, such as physical inactivity and poor eating habits, are essential for the onset and progression of cardiovascular diseases. A social prescription is an approach in which healthcare providers recommend nature-based activities, physical exercise, and healthy living programs to patients with cardiovascular disease as part of their treatment plan.

**Objective:**

This study aimed to investigate the relationship between social prescription of nature, engagement in physical activity, participation in healthy living initiatives, and cardiovascular health, focusing on the mediating role of social support.

**Methods:**

This cross-sectional study was conducted across China. A stratified sampling approach was used to collect primary data from the target population. A total of 5,600 participants were selected from the target population. The collected data were analyzed using structural equation modeling and artificial neural network approaches.

**Results:**

Based on the structural model's bootstrapping, route analysis results demonstrated that each proposed path was significant. The findings revealed strong relationships between these variables, underscoring the importance of social support as a mediator in promoting cardiovascular health through nature exposure, physical activity, and healthy living initiatives. Sensitivity analysis using artificial neural networks revealed that NSP (117.73%) and PaSP (102.50%) were the most significant predictors, followed by HLISP (100%).

**Conclusion:**

Healthcare professionals and policymakers can enhance cardiovascular health outcomes and contribute to the overall well-being of individuals by integrating nature-based interventions, promoting physical activity, and implementing healthy-living initiatives with targeted social support mechanisms.

## 1 Introduction

Population factors, including social, economic, and cultural aspects, education level, and social support, influence health and cardiovascular health outcomes (1). Social prescriptions involve linking patients to other non-clinical services, such as community-based social healthcare practices, and providing food prescriptions that address social determinants of health, thereby enhancing patients' health and well-being (2). SP has been suggested as a measure for evaluating the effects of social circumstances on cardiovascular disease, health behaviors, and potential interventions (3). It is essential to develop strategies to increase health equity and reduce disparities by understanding social determinants of health (SDOH). The effects of social determinants of health encompass cardiovascular and overall health (4, 5). Through multifaceted approaches, interventions targeting health literacy and shared decision-making have positively impacted medication compliance and cardiovascular risk (6). Social prescriptions seek to improve patients‘ health and well-being if they struggle with chronic diseases that are worsened by loneliness (7). Optimizing population health can also be achieved through effective social prescribing and addressing areas other than the biomedical model, such as health promotion and social factors (8). It has become apparent that social prescribing is an effective strategy applicable to various fields, including cardiovascular health. Incorporating self-care and SDOH into social prescriptions improves patient care and resource management (9). These prescriptions can be used as the first or additional measures in solving population health problems, such as cardiovascular health, mental health, and anti-obesity efforts, while considering health inequalities (10).

In particular, social prescriptions can have a meaningful impact on cardiovascular disease. For example, personalized exercise prescriptions have improved specific relevant indicators and risk factors for cardiovascular health and disease (11). Other social prescribing programs, such as nature-related initiatives and farmers' market programs, have been shown to significantly improve patients' cardiovascular health and overall well-being (12). Furthermore, incorporating social support systems and community environments into exercise prescriptions for older adults can enhance the cardiovascular health benefits of exercise prescription programs (13). Social prescribing has been reported to yield multiple benefits for population health and has been linked to better outcomes for obesity, mental health, and health inequalities. Community education has been developed to enhance cardiovascular behavioral profiles (10). Combining tactics for active living into public strategies to promote physical activity and reduce the risks associated with cardiovascular disorders is essential. Supporting habitual exercise in both work activities and other elements of individuals' daily schedules plays a critical role in improving healthy characteristics in society. This approach recommends nature-related activities, such as nature walks, gardening, and conservation efforts, as complementary or supplementary to biomedical treatments (9). There has been an increasing body of empirical literature on the positive impact of nature contact on physical and mental health outcomes, and thus, the inclusion of nature-based activities in interventions such as social prescribing (1, 2).

Social prescribing is a valuable intervention in healthcare, particularly for promoting healthy living and reducing the risk of cardiovascular diseases. It includes linking people with other non-clinical services and functions that exist in the community sector. Various diseases, such as cardiovascular diseases, can be prevented and managed by applying factors such as modifying habits, adopting health-friendly practices, and community endorsement (2, 3). Exercise is essential for cardiovascular health and is considered a key factor in protecting the heart. Muscle-strengthening, endurance, balance, and flexibility exercises are included in cardiac rehabilitation exercise training (14). Physical exercise and nature-oriented therapies can be more effective when combined with social support mechanisms to promote cardiovascular health and overall quality of life. Social support has been shown to act as a mediator of risk factors related to both behavioral and cardiovascular health. Social support serves as a buffer against the effects of natural therapies and physical interventions on cardiovascular health. This practice of social healthcare, which involves engaging community members with patients, has been described and employed to promote healthy aging, cardiovascular health, and social support (2). Active living policies promote social prescribing by enabling people to engage in formal and informal daily physical activities (14). Some networks provide social support, which helps individuals adopt healthy behaviors and enhances their health-related quality of life. Support and resources are key determinants of social prescribing, enabling participation in activities and ultimately promoting health (15).

### 1.1 Theoretical framework

**Figure 1:**
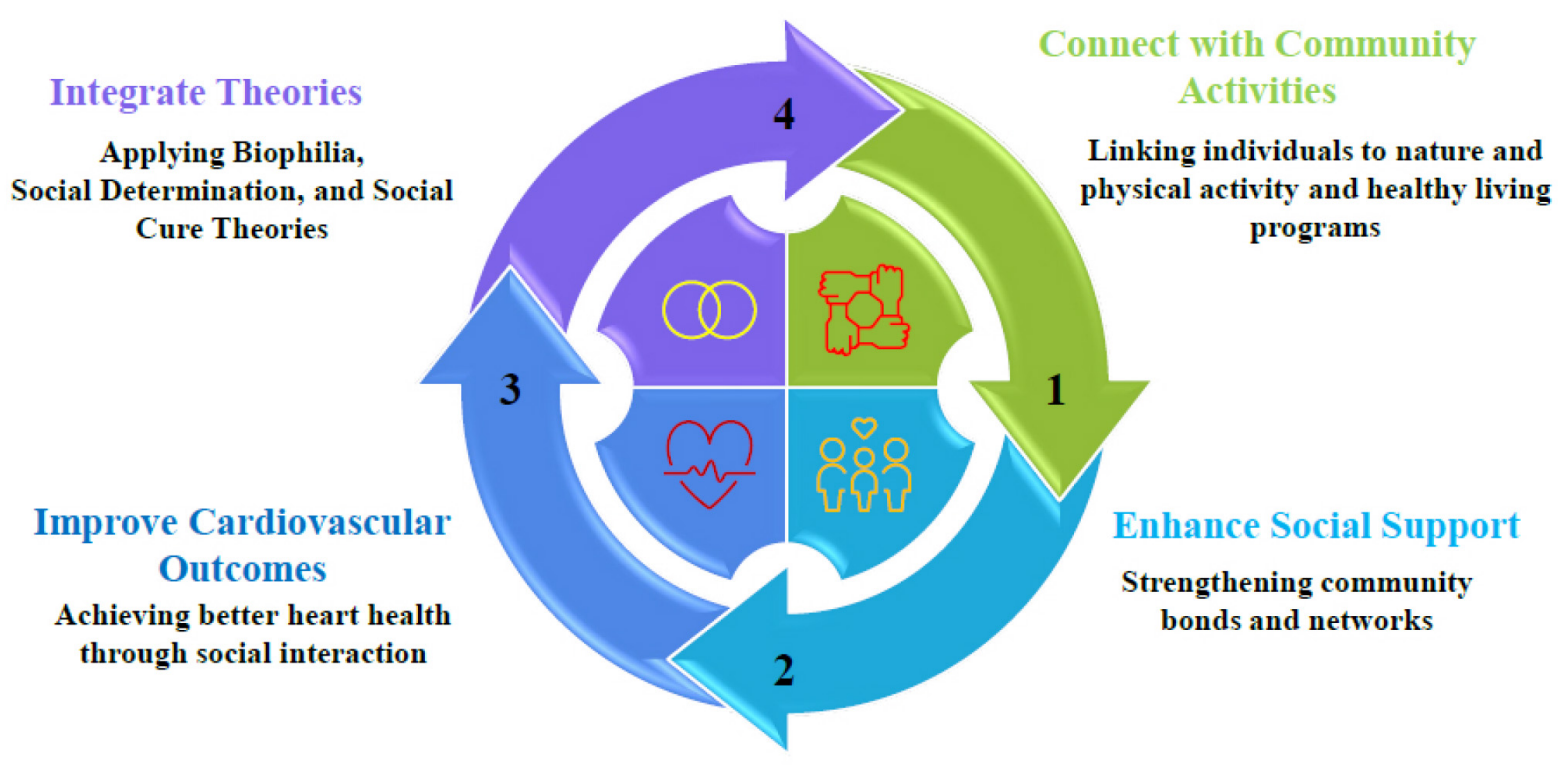
Theoretical framework.

Social Prescription of Nature, Physical Activity (PA), and healthy living initiatives in cardiovascular health offer the potential for non-clinical interventions to enhance cardiovascular outcomes through the mediation of the social support pathway. It is vital to connect people with community-based activities, such as exposure to nature, group exercise, and lifestyle programs, through social prescribing to encourage health enhancement (16). This model integrates the Biophilia Hypothesis (17), Self-Determination Theory (SDT) (18), and Social Cure Theory (19) to demonstrate how interventions lead to cardiovascular benefits through social support. The choice of theories was based on their direct relationship to nature-based interventions, motivational forces of healthy behavior, social processes, and health promotion (20). The model posits that the interventions (independent variables) and cardiovascular health outcomes (dependent variables) are mediated by social support, and the association is reciprocal, such that improved health, in turn, enhances participation in these interventions. The Biophilia Hypothesis advocates for the effectiveness of social prescribing that employs nature as a remedy, presuming that humans have a natural affinity for nature that leads to physiological and psychological benefits. Natural settings, such as green spaces for walking and community gardening, help lower stress biomarkers (e.g., cortisol), improve air quality, and reduce CVD. The situation is further enhanced by these beneficial effects, as group situations contribute to community cohesion and amplify the mediating effect of social support (17). Self-Determination Theory (SDT) defines the success of PA and healthy living programs as intrinsic motivation that relies on autonomy, competence, and relatedness. Prescribed activities, such as group exercises or nutritional workshops, enable participants to make informed choices, learn new skills, and establish social connections. These provisions promote adherence to healthy behaviors, which reduces cardiovascular risk factors, including obesity and sedentary lifestyles, and results in lower blood pressure and lipid profiles. It is the relatedness dimension, in particular, that reinforces social support, as the activities carried out in groups build positive interpersonal relationships that strengthen healthy behaviors (19). The Social Cure Theory views social support as a mediator, positing that participation in an activity group fosters social identity, reduces isolation, and mitigates cardiovascular risk. Social support encompasses emotive, productive, and informational support and emerges within the collective experience of nature-based or PA programs. Robust social networks decrease the risk of cardiovascular death by reducing stress and enhancing adherence to interventions. One key fact is that group nature walks cultivate trust and reduce feelings of loneliness, which in turn lead to improved cardiovascular health through long-term participation (Figure 1) (19, 20).

### 1.2 Statement of the study

**Figure 2:**
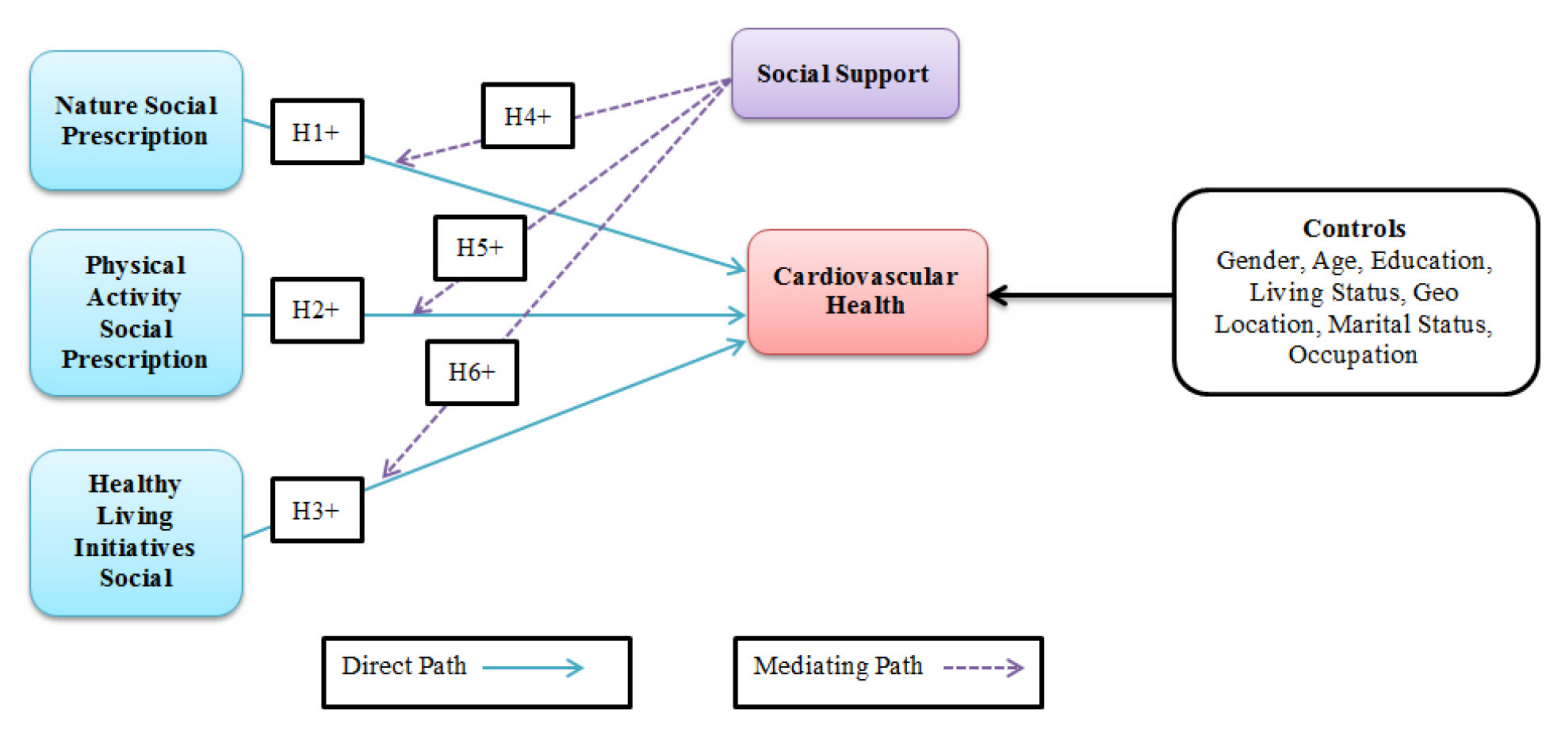
Hypothetical model.

Social prescribing is gaining increasing prominence. Healthcare professionals recommend non-drug treatments for the social determinants of a patient's health. Several social-prescribing methods have been recognized as effective in enhancing physical activity and social engagement among older adults. Cardiovascular diseases (CVDs) are one of the primary causes of mortality in the global population, indicating the need to develop efficient strategies to prevent and treat these diseases. Social prescriptions, particularly when healthcare providers recommend nature-based activities and exercise to patients, are a new and promising method for managing patients' health. Promoting a healthy lifestyle has been established as an essential component of social prescribing, including active living programs and natural prescriptions for health. These programs enable people to incorporate exercise and outdoor activities into their daily lives, positively impacting community health (14). Therefore, physical activity is a well-established determinant of cardiovascular health, as exercise helps individuals reduce their risk of cardiovascular diseases. There has been a development of nature-based social prescriptions that promote the well-being of people by linking them to nature. These initiatives enable humans to connect with the natural environment, ultimately leading to improved cardiovascular health, increased physical activity, and reduced stress levels. Healthy living interventions encompass a range of activities and programs designed to enhance overall quality of life. It encompasses physical, cultural, health, educational, and social dimensions to provide a comprehensive approach to well-being (15). The effectiveness of such interventions in creating positive and sustained shifts in the social determinants of health depends on a more profound understanding of the extent to which contextual factors engage and support individuals undergoing therapy and other prescribed modalities (21). Caregiver support, which encompasses support offered by social networks, is vital for effective health interventions. The essential role of social support in promoting the adoption of appropriate hygiene and health practices in various community contexts has been widely discussed in the literature. Among the antecedents of positive health behaviors, such as exercising, maintaining cleanliness, and practicing personal hygiene, family support stands out as the one that has been consistently identified as influential (22). Consequently, based on the limited knowledge in the literature and exploratory studies by scholars, there is a need to address the growing research gap concerning the vital role of social support in promoting nature, physical activity, and other healthy living initiatives as aspects of social prescriptions that support cardiovascular health. This study examined the relationships between exposure to nature, physical activity levels, participation in healthy lifestyle programs, and cardiovascular health, with social support serving as a moderating factor (Figure 2).

### 1.3 Hypothesis operationalization

Nature-based social prescribing is a relatively new area of focus in healthcare that involves prescribing nature-related activities to improve health (23). Nature prescriptions have been developed to manage diseases and promote physical exercise through participation in activities such as gardening and nature walking (24, 25). These initiatives enhance health and functionality by exposing people to the natural environment and objects, such as plants and gardens (26, 27). Nature-based social prescribing, such as community gardening, requires individuals to engage directly with nature and participate in social activities that improve health outcomes (28). An analysis of the impact of nature-based social prescriptions revealed a positive correlation with improved cardiovascular health. The support of green spaces for exercise, particularly for older individuals, enhances their cardiovascular strength and overall health (29). Nature-based social prescriptions offer substantial cardiovascular health benefits through access to nature, physical activity, and social connectedness. Blue nature-based social prescriptions have been recognized as having strong positive effects on individuals' health and are beneficial for those with chronic diseases (30). The following hypothesis was developed based on the relevant literature.

**H1: nature as a social prescription has a positive effect on cardiovascular health**.

Social prescribing is defined as a model in which general practitioners (GPs) and other health professionals refer patients to additional sources of support to improve their health and well-being (31). Walking, biking, and physical exercise in parks have been found to impact cardiovascular well-being (32). Physical activity has been prescribed more frequently as a social intervention that positively affects cardiovascular health. Exercise training influences the prevention and treatment of cardiovascular diseases, thereby reducing mortality rates (33). Promoting physical activity as part of social prescription aligns well with the social concept of natural prescriptions, wherein green spaces and the natural environment have a positive influence on community health (34). Health promotion or disease prevention models, such as social prescribing programs, have been shown to lower cardiovascular disease risk factors and, consequently, cardiovascular disease incidence, morbidity, and mortality (35). By incorporating physical activity into these programs, individuals can achieve better aerobic fitness levels and other health improvements. Physical activity prescriptions in social prescribing initiatives can also help narrow healthcare access and outcome differences. Personalized exercise prescriptions benefit the objective indexes of cardiovascular health (36). The formulation of the following hypothesis is based on the above literature.

**H2: physical activity as a social prescription has a positive effect on cardiovascular health**.

Several healthy living interventions under social prescriptions have been highlighted for their effectiveness in enhancing cardiovascular health. These innovations aim to promote modifications in diet, exercise, and other stress-related factors to prevent and reduce cardiovascular diseases (37). The American Heart Association focuses on adopting healthy physical behaviors in clinics to improve cardiovascular health (33). Community programs are designed to encourage individuals to make healthier choices and adopt socially and culturally acceptable healthy practices (38). Lifestyle medicine is a sub-discipline that focuses on therapeutic lifestyle changes to manage diseases such as cardiovascular diseases. It focuses on adopting healthy patterns and schedules of exercise and healthy eating, which implies preventing and managing several health issues (39). Moreover, providing free or low-cost food and nutritional information through this intervention also helps promote and enhance cardiovascular health among individuals with diet-related conditions through social prescribing (40). Social prescribing initiatives have immense potential for combating food insecurity and improving dietary practices, which are crucial for maintaining healthy heart function. Increasing the availability of healthy foods leads to improved health standards and reduces the cost of treating heart-related diseases (41, 42). In the population health domain, as seen in the Kuwait National Programme for Healthy Living, promoting healthy living to mitigate the impact of chronic diseases, including cardiovascular diseases (CVDs), is crucial (43). Lifestyle medicine and other community interventions, collectively referred to as healthy living initiatives under social prescriptions, can potentially improve cardiovascular health in diverse populations. The formulation of the following hypothesis is based upon the above pertinent literature.

**H3: healthy living initiatives as social prescriptions are positively associated with cardiovascular health (CVH)**.

Social support reduces cardiac reactivity to stressors and lowers the risk of developing cardiovascular diseases. Perceived social support has been found to reduce cardiovascular reactivity to stress in older adults, a sign of cardiovascular health (44). Social support has been linked to health behaviors, including smoking cessation, exercise, and dietary changes, which impact physical functions, such as the cardiovascular and immune systems (45). Higher social support is associated with lower blood pressure, decreased cardiovascular reactivity, and a more effective functional immune response, whereas loneliness is linked to increased cortisol levels and compromised immunity (46). As applied to cardiovascular health in specific populations, including Black persons and Latinos aged ≥ 45 years and older adults with type 2 diabetes, promoting social support interventions has led to changes in several measures of cardiovascular health (47). Research has shown that social prescribing initiatives, particularly those involving natural environment interventions, can enhance mental health and help manage various medical conditions. Such programs offer individualized services and recreational opportunities that enhance social inclusion, improve psychological and physical well-being, and reduce reliance on medical interventions (48). Social support is beneficial in reducing cardiovascular risk factors, as it offers an opportunity to integrate into society, maintain a good mental state, and influence health-related behavior. Incorporating social support into natural resource-based interventions could enhance cardiovascular health and well-being. The following hypothesis was developed based on the relevant literature.

**H4: social support positively mediates the relationship between nature as a social prescription and cardiovascular health**.

People need to be physically active to support their cardiovascular needs, and exercise is one of the most effective ways to manage and prevent cardiovascular diseases (49). Exercise is generally safe for nearly everyone and offers numerous health and fitness benefits; the risks of exercise decrease as fitness levels improve (2). Coupled with aerobic training, it is recommended for individuals with no cardiovascular disease or those with pre-existing cardiovascular disease to enhance cardiovascular fitness and reduce mortality (50). Vigorous activity that involves endurance training improves endurance and the heart and arteries, whereas high-intensity activities of short and intermittent durations increase muscle strength. The cardiovascular stress response during physical exercise is an early index of cardiovascular fitness, and exercise stress tests are used to identify cardiovascular problems in individuals with cardiac disorders (51, 52). This finding demonstrates that social support is a crucial factor in maintaining cardiovascular health, as numerous studies have shown that it reduces the risk of cardiovascular disease (CVD) and enhances patients' quality of life (53). Healthy living initiatives contribute to this protective mechanism through social support, stress management, exercise, a balanced diet, physiological processes, and improvements in cardiovascular health. Research on social support among clients with chronic diseases, such as coronary artery disease, reveals that self-care is bolstered by peer and family support, indicating the importance of social relationships in addressing cardiovascular diseases (54). As noted above, the positive link between social support, most notably in patients with CVD, has significant implications for the quality and quantity of social networks in enhancing cardiovascular health (55). This study also revealed a positive link between social support and well-being among patients with different CVDs, thus highlighting the importance of social support as a mediator of health in the population (56). Social interactions and cardiovascular health are equally complex, involving social integration, contact, and support, as well as their impacts on health-related behaviors (57). Engaging social networks to publicize and encourage physical activity and assistance can dramatically enhance outcomes for individuals with cardiovascular diseases or those at risk of developing them. The following hypothesis was developed based on the relevant literature.

**H5: the relationship between physical activity as social prescription and cardiovascular health is positively mediated by social support**.

Social support positively impacts self-care, stress management, and quality of life in patients with cardiovascular diseases (58). Individuals with social support exhibit improved coping styles and adhere to healthy living standards, including moderate exercise and a balanced diet, which serve as protective factors for cardiovascular health (59). Furthermore, social support has been proven to cause a decrease in CVD in response to stress, meaning that having a group of people to support you has a physiological advantage. Social support draws more attention to promoting healthy living measures, beginning in childhood. These childhood assets, which are social support, have been associated with favorable cardiovascular health in middle-aged adults (60, 61). This implies that efforts to improve cardiovascular health should include social support approaches in childhood to establish correct preventive values. In addition, peer and family support has been found to increase compliance with self-care measures in patients with chronic illnesses, including CVD, indicating the importance of social connections in managing cardiovascular disease (62, 63). Virtual communities and online support groups are valuable sources of social support for patients with cardiovascular disease. Notably, the support that patients obtain from social health networks regarding information and emotional support can positively affect their health (64). These educational interventions are instrumental in prevention strategies designed to reduce the occurrence of cardiovascular diseases by promoting appropriate and healthy behaviors (65). The most suitable approach for improving cardiovascular health at the clinical level involves training health professionals to deliver healthy living interventions. Lifestyle medicine interventions acknowledge that addressing the social determinants of health is crucial, as life-course approaches emphasize (41, 66). These metrics offer a basis for observing health indicators and outcomes alongside healthy behavioral patterns, with a focus on cardiovascular health and related behavioral risk factors. The following hypothesis was formulated based on the literature:

**H6: Social support positively mediates the relationship between healthy living initiatives, such as social prescriptions, and cardiovascular health**.

## 2 Methods

The study was conducted across the Chinese region among selected respondents using a cross-sectional research design. Ethical approval was obtained from the Fourth Affiliated Hospital Ethics Committee of Zhejiang University International School of Medicine, Yiwu, Zhejiang, China (K2023034).

### 2.1 Sampling and participants

**Figure 3:**
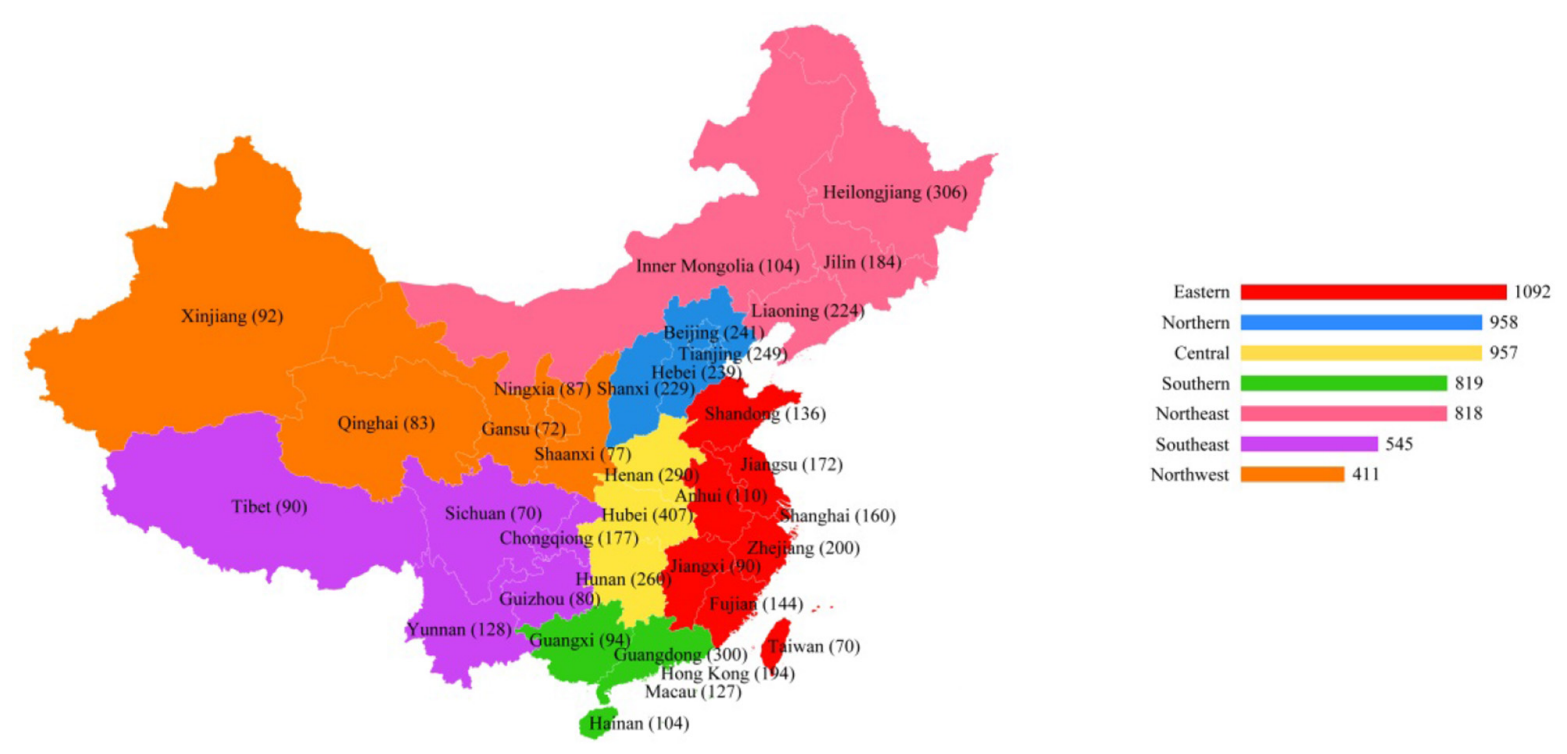
Study population.

China occupies a vast area within the Asia–Pacific region and is endowed with rich geographical and demographic diversity. The study participants were sourced through general practitioners who recommended their patients to non-pharmacological approaches to promote cardiovascular health. A screening criterion was used to identify the target population for the study, ensuring that only qualified participants were included. The inclusion criterion was a predefined age of 33 to 80+ years with cardiovascular health problems who were visiting non-healthcare facilities as part of social prescriptions by general practitioners. A stratified sampling method was adopted to gather primary data from the target populations. Using stratified samples means that each stratum in the population is well represented in the sample. Of the target population, 5,600 participants were selected for the study (See Figure 3).

### 2.2 Data collection instrument

Based on a thorough analysis of the available literature, the instrument was developed as a self-report questionnaire using a five-point Likert scale, consisting of 24 items. Such a scale has been routinely used in the social sciences to measure participants' attitudes from completely disagree to agree. The questionnaire was formulated based on theoretical perspectives and empirical methods, leading to improved content validity and research goal utility. A strict validation protocol has documentary evidence showing that there are no biases to invalidate responses and that the content domain coverage is extensive for the target population. The semi-structured interview technique was employed under the Brislin approach to discuss the questionnaire with ten field experts (67). Two bilingual translators met to jointly change the items, adding the perspectives of ten subject matter experts. Consequently, the need for professional input to ensure that the questionnaires reflect relevant themes in the population under study and consider cultural and contextual differences was considered. After completing the validation stage, a pilot study was conducted in the pre-testing context, involving 27 cases for the application of the questionnaire testing. The exploratory stage was critical in identifying pragmatic problems, such as ambiguous wording, respondent fatigue, and various other logistical issues that might arise during the deployment. The constructs in the questionnaire were checked for internal consistency using Cronbach's alpha, which measures the reliability of individual items based on correlations between them. Alpha values above the conventional threshold of 0.70 were obtained, indicating minimal redundancy/inconsistency between the items (68). Thus, these satisfactory reliability values instilled confidence in using the questionnaire in the main study.

### 2.3 Data collection tool measures

The data collection tools included demographic information, social prescription of physical activity, healthy living initiatives, social support, and cardiovascular health.

#### 2.3.1 Nature social prescription

Social prescriptions consist of healthcare practitioners prescribing time to address and reduce the risk of cardiovascular disease (26). Stress is a chronic condition that increases the risk of developing CVD-related ailments, including hypertension, atherosclerosis, and heart failure. Some studies have noted that time spent in natural settings reduces stress hormones, such as cortisol and adrenaline, thus lowering stress levels (27, 28). Reducing the stress associated with natural exposure is beneficial for managing cardiovascular health. The recommendations provided by Nature for social interactions improve mental health, which is directly related to cardiovascular health (29). Depression, anxiety, and other mental health disorders are also associated with an increased risk of cardiovascular diseases, emphasizing the need for psychological factors in the prevention and treatment of cardiovascular diseases (30, 31). The study participants were asked to provide their perceptions (using a 5-point Likert scale) of the nature of social prescriptions for cardiovascular health.

#### 2.3.2 Physical activity social prescription

Physical activity is an essential intervention for preventing and controlling cardiovascular diseases. Routine walking, hiking, or cycling in parks reciprocates cardiovascular benefits through physical exercise, fresh air and sunlight (32, 33). Exercise in the natural environment has been linked to reduced levels of obesity, diabetes, and other precursors to cardiovascular disease. Hence, it must be part of the framework for preventing and controlling cardiovascular diseases (34). Social prescriptions involving exercise significantly contribute to the prevention of cardiovascular diseases (35). Physical activities benefit heart health by helping the heart muscles increase in strength, lowering blood pressure, reducing high cholesterol, and improving cardiac efficiency. Physical activity promotion includes instances in which a doctor advises a patient on what, when, and how much physical activity to engage in (36, 37). The study participants were requested to report their perceptions (Likert Scale 5-point) regarding physical activity related to social prescription for cardiovascular health.

#### 2.3.3 Healthy living initiatives social prescription

Cardiovascular ailments affect the circulatory system, including the heart and surrounding organs. These conditions are associated with behavioral risks, including lack of exercise, unhealthy eating, tobacco use, and high stress levels (38, 39). By promoting healthy lifestyles that aim to reduce the aforementioned modifiable risk factors, CVD interventions can help reduce the disease burden and improve the overall health of the population (40, 41). Social prescriptions do not employ biomedical approaches but instead incorporate measures such as exercise, nutrition, smoking cessation, and relaxation techniques to address the social determinants that contribute to adverse cardiovascular health. Such programs are typically established by healthcare providers, community/nongovernmental organizations, and public health agencies to enhance cardiovascular health (42, 43). Such approaches are invaluable for healthy living interventions and social considerations in cardiovascular disease (44, 45). The study participants were asked to express their perceptions (using a 5-point Likert scale) of healthy living initiatives and social prescriptions for cardiovascular health.

#### 2.3.4 Social support

Social support is a critical component of cardiovascular health and may enhance the delivery of social prescriptions for people at risk of or diagnosed with cardiovascular diseases (46, 47). By integrating social support mechanisms into social prescriptions, healthcare providers can help patients make sustainable lifestyle changes that improve their quality of life and reduce the risk of future cardiovascular events (CVEs). A component of social prescriptions can be social support interventions to meet the psychosocial needs of patients with cardiovascular conditions (48, 49). Diseases related to the cardiovascular system are not only physical illnesses but also have severe psychological and emotional consequences for patients. Social support systems can consist of companionship, encouragement, and advice, which are crucial for managing the challenges of living with cardiovascular disease (2). Including social support measures in social prescriptions enhances cardiovascular health and decreases the impact of heat stress morbidity on patients and healthcare systems (50). The study participants were requested to provide their perceptions (Likert Scale 5-point) of social prescriptions for cardiovascular health.

#### 2.3.5 Cardiovascular health

The global burden of CVDs remains high, with many more people dying from cardiovascular diseases, contributing to increasing healthcare costs. Social support can facilitate the adoption and sustainability of recommended lifestyle practices for cardiovascular health (41, 47). Social prescriptions can be viewed as integrated by considering the multiple social, economic, and environmental determinants of cardiovascular outcomes (54). Social prescriptions involving nutrition education, meal planning, and improved access to healthy foods can effectively lower CVD risk factors, hypertension, and obesity (58). Healthcare providers‘ role in social prescription for cardiovascular health is to diagnose patients' needs, wants, and circumstances. Social prescriptions for cardiovascular health consider patients as active participants and players in chronic disease management (59). Social prescriptions are a potential way of managing social factors and maximizing CVD outcomes, which involve increasing physical activity, healthy eating, and patient satisfaction with care (60). The study participants were asked to report their perceptions of cardiovascular health (using a 5-point Likert scale) after receiving a social prescription.

### 2.4 Data analysis

The collected data were analyzed using structural equation modeling (SEM) and artificial neural network (ANN) techniques. SEM is a popular method for analyzing multiple relationships between variables, especially for testing the causal relationships among variables. This enables researchers to consider direct and indirect effects in light of theory.

#### 2.4.1 Structural equation modelling (SEM)

The model relies on both factor analysis and multiple regression analyses, allowing for hypothesis testing and theoretical model development. The most important part of SEM is the measurement model, which defines the paths between the observed indicators and the underlying factors (69).

#### 2.4.2 Artificial neural network (ANN)

Neural networks, specifically artificial neural networks (ANNs), have garnered significant attention across a wide range of disciplines, primarily because of their ability to establish relationships and patterns among variables. The diagnostic methods of artificial neural networks involve several necessary steps that should be taken to achieve the effectiveness and reliability of the model. ANNs are mathematical models that emulate the structure and processing of the human brain and consist of discrete, interconnected nodes. ANNs are also a broad process comprising data preparation, model architecture, model training, model assessment, model interpretation, model optimization, and model updating (70). A successful ANN design is a comprehensive and multistep endeavor that is far more complex than merely designing the network architecture. The process begins with data preparation, which involves collecting, cleaning, normalizing, and dividing the data into training, validation, and test sets. This initial step has a profound impact on the final performance of the model. Second, the model structure is specified, determining the number of layers, nodes, and connection types (e.g., convolutional for images and recurrent for sequences) utilized. The model is trained, where thee network is educated by iteratively changing weights using an algorithm. Once trained, the model is heavily tested on held-out test data to calculate its accuracy, precision, and other performance metrics. Interpretation methods are then applied to understand how the model arrives at its conclusions, which is essential for establishing trust and identifying potential biases in the model. Model optimization (or hyperparameter tuning) is performed based on the analysis to maximize performance (70).

## 3 Results

### 3.1 Demographic information of the participants

Table 1 shows the demographic information of the study participants. The gender distribution shows that the majority of participants were female (38.7 %), followed by males (25.7 %), with a significant portion (35.6 %) preferring not to disclose their gender. In terms of age, the largest group was between 73–82 years old, comprising 29.3% of the total, while the youngest group, aged 33–42, makes up 10.4%. The educational background is diverse, with the highest percentage of participants having a college degree (28.7 %) and the lowest percentage having primary education (26.5 %). Living status reveals that a slight majority, 39.8%, live in other unspecified arrangements, while 31.4% live alone and 28.8% live with family. Geographically, the participants were spread across China, with the highest concentration in the Eastern region at 21.8%. Regarding marital status, a significant number of participants were single (35.2 %), and a considerable portion were divorced (28.2 %). Regarding occupation, the most represented group was retirees at 20.4%, with government employees and the unemployed following closely at 15.3% and 17.4%, respectively.

**Table 1:** Demographic information of the participants (*N*-5,600).

**Variables**	**Categories**	**Frequency/Percentage**
Gender	Male	1,432 (25.7%)
	Female	2,158 (38.2%)
	Prefer not to answer	2,010 (35.9%)
Age	33–42	585 (10.4%)
	43–52	680 (12.1%)
	53–62	832 (14.9%)
	63–72	1,108 (19.8%)
	73–82	1,642 (29.32%)
	+83	753 (13.4%)
Education	Primary	1,482 (26.5%)
	Secondary	1,359 (24.3%)
	College graduate	1,607 (28.7%)
	University graduate	876 (15.6%)
	Other	276 (4.9%)
Living status	Live alone	1,760 (31.4%)
	Live with family	1,612 (28.8%)
	Other	2,228 (39.8%)
Geo location	Northern China	1,174 (21.0%)
	Eastern China	1,041(18.6%)
	Central China	1,147 (20.5%)
	Southern China	1,222 (21.8%)
	Northeast China	1,016 (18.1%)
Marital status	Single	1,971(35.2%)
	Married	969 (17.3%)
	Divorced	1579 (28.2%)
	Widowed	1081(19.3%)
Occupation	Government employee	858 (15.3%)
	Unemployed	973 (17.4%)
	Self-employed	935 (16.7%)
	Private-employed	922 (16.5%)
	Retired	1,144 (20.4%)
	Other	768 (13.7%)

### 3.2 Non-linear relationships

Hence, we opted for the SEM-ANN method, as factor-based and composite-based SEMs cannot handle non-linear relationships, given the evidence of non-linearity (see Table 2). The positive and negative non-linear correlations in our model were as follows:

**Table 2:** ANOVA.

**Variables**	**SS**	**DF**	**MS**	**F**	**S**	**L**
HLISP^*^CH	1,591.775	172	9.255	12.522	0.000	No
NSP^*^CH	1,459.425	172	8.485	11.110	0.000	No
PaSP^*^CH	1,413.565	172	8.218	10.657	0.000	No

SS, sum of square; DF, degree of freedom; MS, mean square; F, F statistical test; S, significant; L, linear.

### 3.3 Common method bias (CMB)

The heterotrait-monotrait (HTMT) and internal variance inflation factor (VIF) were applied during the examination of CMB. CMB occurs when the significant constructs are highly inter-correlated (correlation coefficient > 0.90). All correlation values for the study constructs were <0.90. Therefore, there is no CMB because the maximum correlation value is zero. The maximum VIF for the current study is 1.870, which is less than the threshold value of 3.30, suggesting no concern regarding CMB (71).

### 3.4 Model measurement

**Figure 4:**
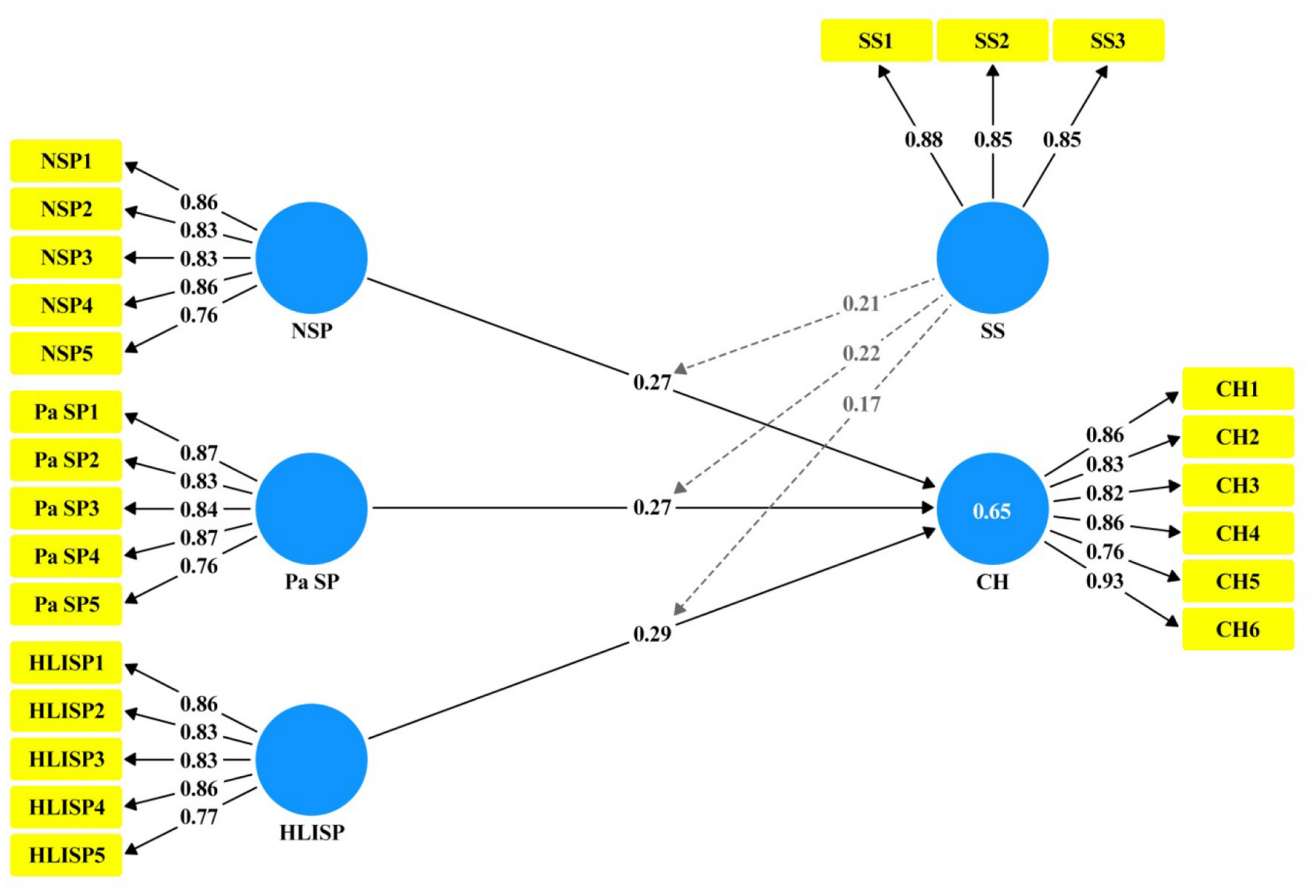
Model measurement.

A measurement model was established using Smart-PLS software 4.0, which revealed the factor loadings (FL) of all items and found that all were within the interval of 0. After confirming that all threshold values had been reached, we proceeded to the second phase of the structural model to analyze whether the proposed hypotheses were true (69). The average variance extracted (AVE) values obtained were >0. The convergent validity of all items regarding their respective constructs was confirmed. The multicollinearity of all items was assessed using the variation influence factor (VIF) value. The Cronbach's alpha (CA) and composite reliability (CR) were >0.70, indicating good internal consistency (see Table 3 and Figure 4).

**Table 3:** Construct validity and reliability.

**Construct**	**Items**	**factor loading**	**VIF**	**Cronbach's Alpha**	**Composite reliability**	**Average variance extracted**
Cardiovascular health	CH1	0.86	2.783	0.92	0.92	0.71
	CH2	0.83	2.451			
	CH3	0.82	2.362			
	CH4	0.86	2.920			
	CH5	0.76	1.793			
	CH6	0.93	4.884			
Healthy living initiatives social prescription	HLISP1	0.86	2.485	0.89	0.89	0.69
	HLISP2	0.83	2.213			
	HLISP3	0.83	2.163			
	HLISP4	0.86	2.615			
	HLISP5	0.77	1.729			
Nature social prescription	NSP1	0.86	2.460	0.88	0.89	0.69
	NSP2	0.83	2.140			
	NSP3	0.83	2.135			
	NSP4	0.86	2.536			
	NSP5	0.76	1.673			
Physical activity social prescription	PaSP1	0.87	2.596	0.89	0.92	0.69
	PaSP2	0.83	2.206			
	PaSP03	0.84	2.167			
	PaSP4	0.87	2.689			
	PaSP5	0.76	1.695			
Social support	SS1	0.88	2.058	0.83	0.90	0.75
	SS2	0.85	1.902			
	SS3	0.85	1.792			

### 3.5 Discriminant validity test

Discriminant validity was further tested using the Fornell-Larcker test and the HTMT ratios of herbivores and mono-traits (Tables 4, 5). The findings of the Fornell-Larcker (72) analysis consist of two approximately square matrices below the diagonal matrix of the constructs' correlations. The square root of its AVE measures that each construct's reliability is greater than the correlations below its diagonal, establishing discriminant validity. All correlation values were <0.9, confirming discriminant validity.

**Table 4:** Fornell–Larcker criterion values.

**Constructs**	**CH**	**HLISP**	**NSP**	**PaSP**	**SS**
CH	0.84				
HLISP	0.44	0.83			
NSP	0.42	0.28	0.83		
PaSP	0.42	0.28	0.35	0.83	
SS	0.44	0.42	0.27	0.27	0.86

CH, cardiovascular health; HLISP, healthy living initiatives social prescription; NSP, nature social prescription; PaSP, physical activity social prescription; SS, social support.

**Table 5:** HTMT values.

**Constructs**	**CH**	**HLISP**	**NSP**	**PaSP**	**SS**	SS ^*^ HLISP	SS ^*^ NSP	SS^*^ PaSP
CH								
HLISP	0.49							
NSP	0.47	0.32						
PaSP	0.46	0.31	0.39					
SS	0.50	0.49	0.31	0.32				
SS^*^HLISP	0.18	0.24	0.17	0.18	0.25			
SS^*^NSP	0.27	0.18	0.14	0.19	0.19	0.40		
SS^*^PaSP	0.27	0.18	0.18	0.16	0.19	0.39	0.48	—

CH, cardiovascular health; HLISP, healthy living initiatives social prescription; NSP, nature social prescription; PaSP, physical activity social prescription; SS, social support.

### 3.6 Assessment of the structural model

When examining the structural model, we first analyzed the inner variance inflation factor (VIF) to check for collinearity. Once the VIF was computed again, we found that the VIF value was below 3, which is less than 5; hence, it was agreed that there was no collinearity issue. Nevertheless, the test analysis is in the form of the magnitude of the effect size, which is (*F*^2^), and the coefficient of determination (*R*^2)^. All these values are greater than or equal to the threshold values listed in Table 6.

**Table 6:** Assessment of the structural model.

**Statistical tests**	**R-square**	**Adjusted r-square**	**Criteria**
*R* ^2^	0.65	0.65	0.26: Substantial, 0.13: Moderate, 0.02: Weak
**Endogenous variables**	**Adjusted R-square**		
*F* ^2^	CH	–	0.26: Substantial, 0.13: Moderate, 0.02: Weak
	HLISP	0.19	
	NSP	0.17	
	PaSP	0.18	
	SS	0.19	
	SS^*^HLISP	0.09	
	SS^*^NSP	0.11	
Collinearity	CH	–	VIF < = 5.0
	HLISP	1.31	
	NSP	1.22	
	PaSP	1.22	
	SS	1.30	
	SS^*^HLISP	1.32	
	SS^*^NSP	1.41	
	SS^*^PaSP	1.40	

*R*^2^, coefficient of determination; AR^2^, adjusted coefficient of determination; *F*^2^, Effect Size; CH, cardiovascular health; HLISP, healthy living initiatives social prescription; NSP, nature social prescription; PaSP, physical activity social prescription; SS, social support.

### 3.7 Model fitness

The model fitness in this study was examined using the standardized root mean square residual (SRMR), normed fit index (NFI), and chi-square (χ2) values. SRMR value is a standardized-residual index that was developed among observed covariance and hypothesized matrices, which shows the measurement of model fitness (see Table 7). The acceptable range of the SRMR value is less than or equal to 0.08. According to the results, the estimated SRMR value was 0.033, which is acceptable as a good model fit. The NFI value is 0.946, and chi-square (χ2) shows the value of 4335.985.

**Table 7:** Model fit summary.

**Fit Indices**	**Estimated model**
SRMR	0.033
d_ULS	0.337
d_G	0.133
Chi-square	4335.985
NFI	0.946

SRMR, standardized-root-mean-square-residual; d_ULS, unweighted least squares discrepancy; d_G, geodesic distance; NFI, normed fit index.

### 3.8 Hypothesis testing

**Figure 5:**
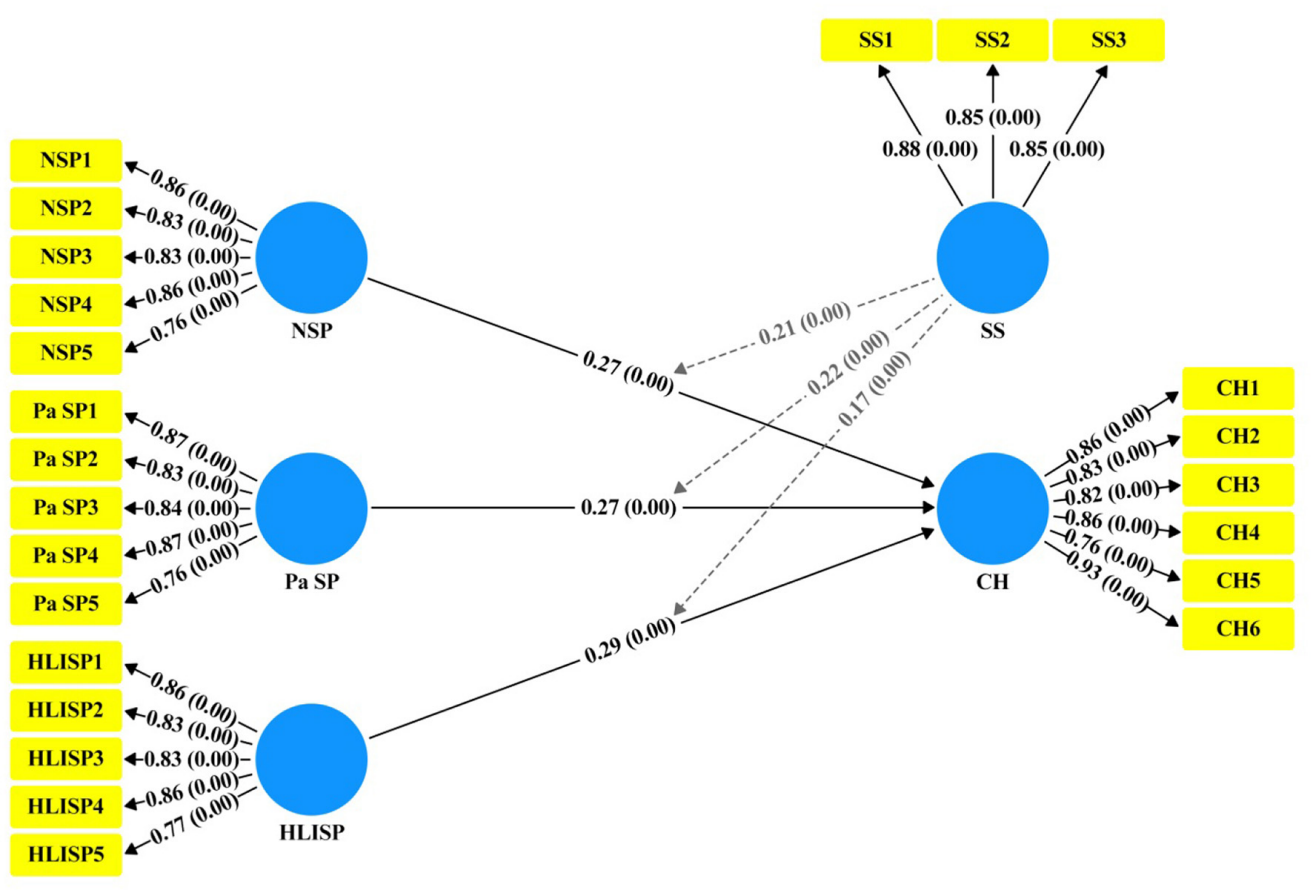
Path analysis.

We utilized both 95% confidence intervals and t-statistics to determine the significance of these associations. The results of the structural model bootstrapping (see Figure 5, Table 8) shows that each path is significant (H1: β = 0.27, CI = 95%, LL = 0.250, UL = 0.290, *t* = 26.81, *p* = 0.00); (H2: β = 0.27, CI = 95%, LL = 0.250, UL = 0.290, *t* = 27.64, *p* = 0.00); (H3: β = 0.29, CI = 95%, LL = 0.270, UL = 0.320, *t* = 26.94, *p* = 0.00); (H4: β = 0.21, CI = 95%, LL = 0.190, UL = 0.230, t = 19.88, *p* = 0.00); (H5: β = 0.22, CI = 95%, LL = 0.200, UL = 0.240, t =21.29, *p* = 0.00); (H6: β = 0.17, CI = 95%, LL = 0.150, UL = 0.1900, *t* = 17.68, *p* = 0.00).

**Table 8:** Hypothesis testing results.

**Hypotheses**	**OS/Beta**	**Confidence interval 95% bias corrected**		***t* value**	***p* value**	**Decision**
		**LL**	**UL**			
NSP -> CH (H1)	0.27	0.250	0.290	26.81	0.00	Supported
PaSP -> CH (H2)	0.27	0.250	0.290	27.64	0.00	Supported
HLISP -> CH (H3)	0.29	0.270	0.320	26.94	0.00	Supported
SS^*^NSP -> CH (H4)	0.21	0.190	0.230	19.88	0.00	Supported
SS^*^PaSP -> CH (H5)	0.22	0.200	0.240	21.29	0.00	Supported
SS^*^HLISP -> CH (H6)	0.17	0.150	0.190	17.68	0.00	Supported

CH, cardiovascular health; HLISP, healthy living initiatives social prescription; NSP, nature social prescription; PaSP, physical activity social prescription; SS, social support.

### 3.9 Artificial neural network analysis

Multilayer artificial neural networks with input, hidden, and output layers were selected for the applications. During the analysis process, we verified the data fitness and calculated the root mean square of the errors. One of the relevant features of the employed ANN architecture is the complexity and structure of the output neuron, which has two hidden layers. The sample usage was divided into training and testing sets, with 90% of the samples allocated for training and the remaining 10% for testing (Table 9). The accuracy of the ANN prediction model was verified using the root mean square error (RMSE) in both the training and testing datasets (73). The average RMSEs of the training and testing data are 0.5878 and 0.5838, respectively, as shown in the last two columns of Table 8. Thus, to confirm the previous fact, it can be concluded that the model fits the data well.

**Table 9:** RMSE values.

**Training**			**Testing**			**Total Samples**
***N***	**SSE**	**RMSE**	***N***	**SSE**	**RMSE**	
5,062	1,450.495	0.535299767	538	159.845	0.5451	5,600
5,049	1,289.592	0.505386324	551	138.606	0.5016	5,600
4,992	1,393.056	0.52825918	608	151.377	0.4990	5,600
4,975	1,396.347	0.529785625	625	165.013	0.5138	5,600
5,061	1,282.166	0.503331303	539	130.755	0.4925	5,600
5,007	1,418.757	0.532310666	593	153.364	0.5086	5,600
5,043	1,340.383	0.515549059	557	114.208	0.4528	5,600
5,003	1,275.493	0.504921457	597	127.721	0.4625	5,600
5,016	1,438.982	0.535610296	584	181.174	0.5570	5,600
5,050	1,336.616	0.514467194	550	141.362	0.5070	5,600
5,062	1,450.495	0.535299767	538	159.845	0.5451	5,600
5,049	1,289.592	0.505386324	551	138.606	0.5016	5,600
Mean	1,363.498	0.520467	Mean	147.57	0.507217	
SD	66.0694	0.012934	SD	17.53935	0.029863	

*N*, number; SSE, standard square error; RMSE, root mean square error.

### 3.10 Sensitivity analysis

Consequently, the relative importance of the above results was obtained by dividing them by their maximum significance. The results are presented as percentages to determine the predictive power of each input neuron desirability factor (Table 10). The most significant predictors are HLISP (Healthy Living Initiative Social Prescription (HLISP) at 100%, Nature Social Prescription (NSP) at 117.73%, and Physical Activity Social Prescription (Pa SP) at 102.50%. The normalized importance percentages for different Artificial Neural Network (ANN) configurations, including ANN (i) through ANN (x), with NSP demonstrating the highest normalized importance at 117.73%, followed by Pa SP at 102.50%, and HLISP at 100%. This indicates that NSP has the greatest influence on the predictive model, followed closely by Pa SP and HLISP, providing a comprehensive understanding of the key variables driving this analysis.

**Table 10:** Sensitivity analysis.

**ANN**	**NSP**	**Pa SP**	**HLISP**
ANN (i)	1.00	0.79	0.82
ANN (ii)	1.00	0.88	0.87
ANN (iii)	1.00	0.92	0.76
ANN (iv)	1.00	0.80	0.84
ANN (v)	0.98	1.00	0.88
ANN (vi)	1.00	0.85	0.81
ANN (vii)	1.00	0.88	0.81
ANN (viii)	0.93	0.96	1.00
ANN (ix)	1.00	0.80	0.79
ANN (x)	1.00	0.75	0.84
Average Importance	0.92	0.89	0.88
Normalized importance (%)	117.73%	102.50%	100%

ANN, artificial neural network; NSP, nature social prescription; PaSP, physical activity social prescription; HLISP, healthy living initiative social prescription.

## 4 Discussions

The present study employed structural equation modelling (SEM) and artificial neural network (ANN) design to examine the relationship between social prescriptions to nature (NSP), physical activity (PaSP), and healthy living initiatives (HLISP) and cardiovascular health (CH), mediated by social support (SS). Social prescription is recognized as a practical approach in health systems that prioritize patient healing through non-traditional medical interventions, focusing on personal well-being (74). The results fully support all hypothesized hypotheses and note the significant direct and mediated effects of these social prescriptions on CH, with a large population of Chinese people with cardiovascular issues. The results align with and build upon previous research on social prescribing as a non-pharmacological intervention for managing cardiovascular diseases, emphasizing its potential to improve health equity and well-being (2, 10). Lifestyle alterations are an essential component of preventive strategies that can be effectively integrated into the fight against cardiovascular diseases. Learning optimal CVD and heart health practices may involve adopting a healthier lifestyle, which includes quitting smoking or not starting smoking, eating a nutritious and balanced diet, exercising regularly, and other similar practices (38). Autonomy support and patient self-regulation are crucial for achieving desirable health effects, particularly in individuals with heart illnesses (75).

Green social prescribing has recently been identified as a highly effective method for improving cardiovascular health and promoting well-being (2). Nature-based social prescriptions can help reduce health inequalities because of their low cost and ease of access, particularly in areas of deprivation that tend to exhibit higher health disparities (76). Social prescribing has progressed in nature-based solutions that entail natural sources from horticulture, related actions to conserve species, and activities resulting from natural solutions intricately linked with community health interventions for managing NCDs, mental health challenges, and social exclusion (27). The analysis confirmed that social prescription components and CH were in an intensive, direct, and positive relationship. Specifically, NSP positively influenced CH (H1: β = 0.27, *p* < 0.001), particularly because it added therapeutic value to nature-based activities (gardening, nature walks, and exposure to green spaces) for enhancing cardiovascular outcomes. This evidence confirms the earlier literature, which shows that nature prescriptions lower stress hormones, such as cortisol, improve mental health, and reduce CVD risk factors by enhancing physical activity and social connectedness (18–25). Nature prescription programs have been widely accepted and implemented to address the modern high incidence of chronic diseases and physical inactivity caused by unhealthy lifestyles of participants (19). These programs, for example, “blue prescriptions,” have been linked to enhancing the health of different people, particularly those with chronic health conditions (25). Nature-based interventions are typically planned as social activities that incorporate strategies to address the lack of social inclusion and low community participation (77). These interventions are related to staff physical and psychological health, with research evidence showing that they have a significantly positive effect on staff self-reported well-being and heart rate variability (78).

The positive effects of exercise include enhanced cardiovascular function and dynamic changes, prevention of cardiomyopathies, augmentation of cardiac reserve, and regulation of autonomic activity. Physical activity is one of the most effective ways to promote cardiovascular health (CVH). Additionally, exercise is beneficial for protecting against cardiovascular diseases and their occurrence, as well as for treating patients with such diseases. It can also be a form of therapy involving nonsurgical treatment, which is recommended to enhance cardiovascular fitness and function (79). Similarly, PaSP also revealed a direct effect (H2: β = 0.27, *p* < 0.001), which validates the recognized direct impact of prescribed physical activities that involve walking, bicycling, or organized exercise to prevent the development of CVD, improve aerobic fitness, and reduce mortality rates (2, 5, 11, 26–31, 46–49). H3: β = 0.29, *p* < 0.001, had the highest direct relationship with HLISP. Programs that enhance balanced diets, smoking prevention, and stress prevention are instrumental in addressing modifiable CVD risk factors (32–39). Exercise training has been categorized as the first level, aiming to prevent further cardiovascular disease morbidity and mortality (80). Cross-sectional epidemiological and intervention reviews have suggested that exercise may reduce the risk of cardiovascular and metabolic diseases. Aerobic exercise training is one of the five core components for people with cardiovascular diseases; therefore, supporting such cardiac rehabilitation practices is warranted. Aerobic exercise training benefits the cardiovascular system, especially in specific groups, such as postmenopausal women with hypertension (81, 82).

Youth well-being, which has been investigated in Brazil, Colombia, and Mexico, illustrates different ways to achieve cardiovascular health, ranging from exercise-focused paradigms to training new sustainable healthy habits and prolonging the health span of the interventions. This finding reinforces thee earlier call to address these factors early and highlights the importance of utilizing multiple approaches and methods to promote healthy living (83). The most significant contribution of this study is the explanation of SS as a mediator of these relationships. SS mediated the positive effect of NSP on CH (H4: β = 0.21, *p* < 0.001), such that social networks mediate the cardiovascular benefits of exposure to nature by supporting emotions, reducing isolation, and stimulating long-term engagement (50–54). The idea behind social prescribing is that there is a connection between health and social conditions, such as social interaction, access to healthy food, and engagement in sports (84). This intermediary effect aligns with the existing literature on the buffering effects of social support, stress impacts, and nature's facilitating role. Similarly, SS mediated the relationship between PaSP and CH (H5: β = 0.22, *p* < 0.001), the strongest mediated relationship, which supports the fact that family and peer support positively influences the cardio-protective effects of exercise by improving self-care behaviors and resiliency in patients with CVD (41, 50–54, 73–77). Finally, SS mediated the connection between HLISP and CH (H6: β = 0.17, *p* < 0.001), despite having a negligible effect, as social support aids compliance with healthy living behaviors, such as dietary change and stress management, resulting in long-term cardiovascular outcomes (41, 55–61, 78, 79). These mechanisms support the view that the efficacy of social prescribing is enhanced by its integration into supportive community contexts and consideration of psychosocial determinants of CVD (8).

## 5 Conclusion

The protective effects on the heart and the consequent beneficial impact on long-term risk factors and outcomes underscore the importance of regular exercise. Exercise recommendations should be individualized based on the activity type, duration, and intensity to maximize the cardio-protective effects of exercise. Clinical, community, and individual factors must be balanced to promote healthy living policies and practices in the treatment of cardiovascular disease. Different populations and settings result in various social prescriptions, personalized care strategies, and lifestyle changes that effectively manage the risk of cardiovascular disease. Nature-based social prescriptions are an innovative approach to advancing and promoting cardiovascular health through nature, social frameworks, and comprehensive health strategies. These approaches effectively eliminate health inequalities and improve patients' perceived recovery and overall cardiovascular health outcomes through nature, which is a healing process. Physical activity remains the cornerstone of the treatment and prevention of cardiovascular diseases.

### 5.1 Policy implications for public health practitioners

The results of this study are helpful for public health practitioners interested in the health of the CVDs population and the use of nonmedical integrative interventions for health promotion. Social prescriptions (i.e., nature-based physical activity and mass healthy living programmes) should be offered as standard-of-care for clinical and community-based practice. They must be included in a broader health policy. Interventions like these should be focused on people who are at high risk of developing CVD or who have problems coping with their disease. Thus, structural components of interventions associated with social networks, such as the formation of community circles for training and peer support, are recommended to act as mediators for increased efficiency of prescriptions and compliance by long-term patients. Some health professionals are encouraged to work with primary care physicians and community development regarding social determinants of health to decrease health disparities and increase referrals and access to interventions. Furthermore, medical professionals' training in the practice of lifestyle medicine and social prescribing in countries beyond China should also benefit from the establishment of appropriate policy and practice frameworks to enable and disseminate mainstream social evidence-based interventions. To optimize these strategies for decreasing CV morbidity and mortality at the population level, outcomes and participation rates must be monitored.

### 5.2 Study limitations

The current investigation has several limitations: the cross-sectional nature of the design and the data gathered through self-administered questionnaires. Additionally, the study sample had certain restrictions in terms of demographics and geographical location, which may make the findings less applicable to other demographic groups or cultural settings. The inclusion of perceived social support as a mediator meant that other possible moderating variables, such as gender and comorbid conditions, were not explored. Future studies are needed to examine these moderators to detect the limits of these relationships.

## Data Availability

The raw data supporting the conclusions of this article will be made available by the authors, without undue reservation.
